# Research status of fetal hydrocephalus from 2003 to 2022 based on bibliometric analysis

**DOI:** 10.1002/ibra.12171

**Published:** 2024-08-18

**Authors:** Qian Li, Zheyu Song, Chenyang Zhai, Sajid Hussain, Wenxue Zhao, Shunwu Xiao

**Affiliations:** ^1^ Department of Neurosurgery Affiliated Hospital of Zunyi Medical University Zunyi China; ^2^ The First Clinical Institute Zunyi Medical University Zunyi China; ^3^ Institute of Neuroscience Kunming Medical University Kunming China; ^4^ School of Applied Sciences and Humanities National University of Technology Islamabad Pakistan

**Keywords:** bibliometric analysis, congenital, human fetus, hydrocephalus

## Abstract

Hydrocephalus is the most common and devastating condition affecting the fetus. The aim of this study was to provide a comprehensive overview of the relevant literature through bibliometric analysis. The survey covers the articles related to congenital hydrocephalus published in the Web of Science Core Collection (WoSCC) database from January 1, 2003 to December 31, 2022. In addition to repeated literature, reviews and articles are included. We visualized the annual publication number, citation frequency, country/region, institution, author, periodical, and keywords with a range of software such as VOSviewer (1.6.18), Microsoft Excel 2019 (Redmond) and online analysis platform (https://bibliometric.com/ document). The results showed that the United States made the most important contribution to the research on fetal hydrocephalus. China's contribution has grown and developed strongly in recent years. The key words were mainly divided into four categories: basic research, epidemiology, treatment, and diagnostics. The number of publications related to fetal hydrocephalus has increased significantly, and it has a good development prospect in prenatal diagnosis and treatment.

## Graphical abstract

In this study, we extracted 613 articles related to hydrocephalus from the Web of Science Core Collection (WOSCC) database published from January 01, 2003 to December 31, 2022, and 613 articles were obtained after weight reduction using CiteSpace. CiteSpace and VOSviewer software were used to extract and visualize the elements of the article, including the number of annual publications, citation frequency, country, institution, author, and journal keywords.

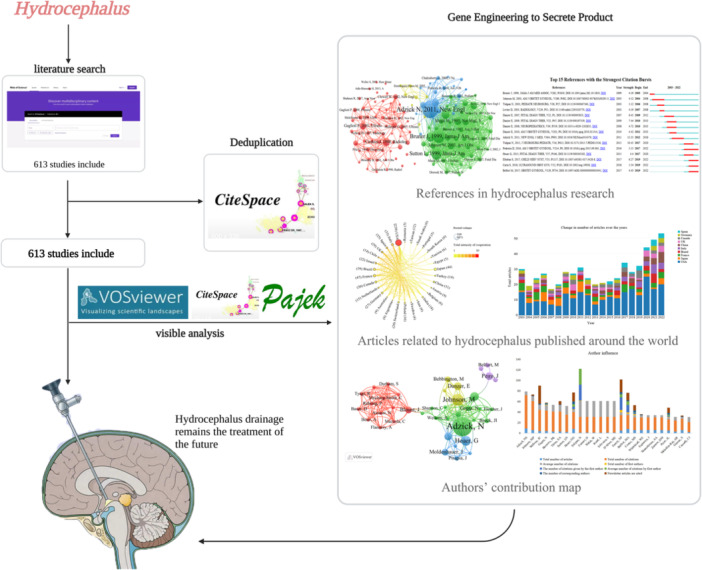

## 1 INTRODUCTION

Fetal hydrocephalus is one of the most common congenital malformations of the central nervous system.^1^ The prevalence rate is about 1.1 cases per 1000 newborns, and it is estimated that one newborn in every 500–1000 newborns in the United States suffers from fetal hydrocephalus.^2^, ^3^ This condition is characterized by the pathological accumulation of cerebrospinal fluid (CSF), leading to ventricular enlargement. Various etiologies can cause fetal hydrocephalus, with spina bifida (myelomeningocele), aqueductal stenosis, and Dandy‐Walker malformation being among the most common.^4^, ^5^ It often occurs with such physical symptoms as Dome‐shaped skull, persistent fontanelle, and bilateral ventrolateral strabismus.^6^ Fetal hydrocephalus is not only a damaged disease of the “brain canal” that can be treated by CSF metastasis, but also a complex neurodevelopmental disorder with related functional defects that can be referred to the brain parenchyma.^7^ Additionally, genetic factors play a significant role, with hereditary causes accounting for up to 50% of cases.^8^

Bibliometric analysis refers to the quantitative analysis of published articles on the topic of interest through statistics and mathematics.^9^ It shows the relationship between research and publication year, citation times, author, institution, and country.^10^ At present, it has been widely applied to macroscopic observation and consideration of various fields and disciplines.^11^, ^12^ Recently, graphical tools have been updated to visualize the results of big data, and bibliometric analysis has been applied to various medical fields related to autoimmune diseases (such as psoriatic arthritis) in the field of complementary medicine.^13^ However, it has not been implemented in the field of hydrocephalus. Because fetal hydrocephalus is one of the important diseases affecting human health, it is of great significance for us to explore its research progress.

The purpose of this study was to conduct a bibliometric analysis of fetal hydrocephalus studies from 2003 to 2022 based on all published studies with the usage of the Web of Science Core Collection (WoSCC) database to analyze study trends. Based on the symbiotic network of countries, institutions, authors, journals, and keyword clustering from 2003 to 2022, this study provided insights into the literature of fetal hydrocephalus. By analyzing the information in the visual network, this paper discussed the research hotspots and frontier trends in the field of fetal hydrocephalus, so that scholars can quickly grasp the research direction and scope of related fields.

## 2 METHODS

### 2.1 Data collection and screening

**Figure 1:**
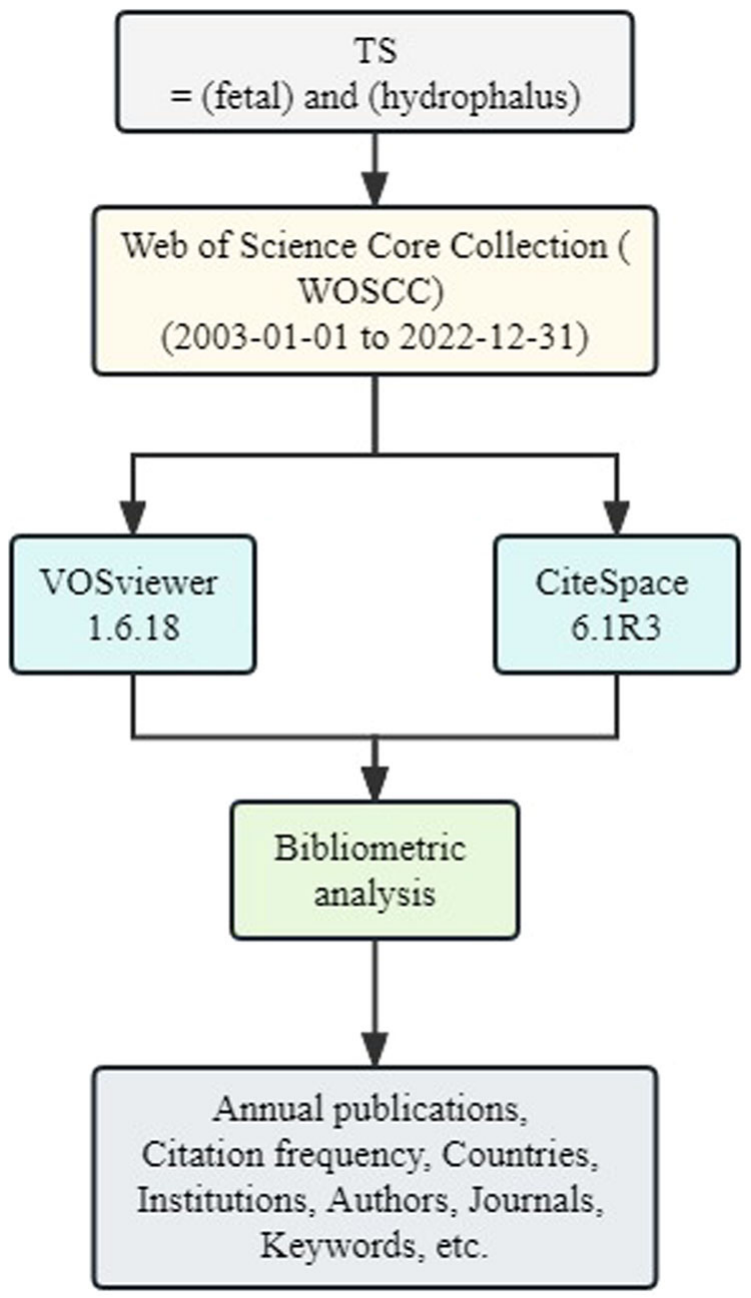
Flow chart. [Color figure can be viewed at wileyonlinelibrary.com]

The WoSCC is considered one of the most commonly used databases for bibliometric research.^14^ In this study, we extracted articles related to fetal hydrocephalus from the WoSCC databases published from January 01, 2003 to December 31, 2022. A search was performed using “TS = (fetal) and (hydrophalus)” and all retrieved records were downloaded in the “txt” format. Only articles and reviews in English were selected. Literature selection and data extraction were performed by two independent researchers to ensure the reliability of the results. To ensure the comprehensive and reliable results, the researchers deleted the repeated literature and included all the other relevant literature results. Elements were extracted and analyzed from selected articles, including data on number of annual publications, citation frequency, country/region, institution, author, journal, and keywords. We showed the screening process through a flow chart (Figure 1).

### 2.2 Literature quantitative analysis

VOSviewer 1.6.18 (downloaded from https://www.vosviewer.com/) is widely used to build and visualize networks based on scientific publications, scientific journals, researchers, research organizations, countries, or keywords. In this study, we used the software for country, magazine, co‐author analysis, keyword co‐occurrence analysis, and reference co‐citation analysis. We deduplicated the data before performing the analysis. In a network produced by VOSviewer, the node size represents the number of publications, and the larger node represents the larger number of publications. Links between nodes represent correlations between parameters (country, institution, author, or keyword), and the thickness of the links represents the correlation strength. As mentioned, the importance of a node in a network is determined quantitatively by its total link strength (TLS) with other nodes. For keyword cluster analysis, each color on that graph represents a keyword category.

In addition to the VOSviewer, CiteSpace 6.1.R3 is also used as an important scientific measurement tool for analyzing active areas and trends in research within the scientific community. In this study, CiteSpace was used to construct and visualize the first 15 most frequently cited references.

Besides, Microsoft Excel 2019 (Microsoft Corporation) and Bibliometric Online Analysis Platform (https://bibliometric.com/) were also used for data analysis and visualization, such as annual volume of published articles, and national status of published articles.

## 3 RESULT

### 3.1 Annual publications since 2003

**Figure 2:**
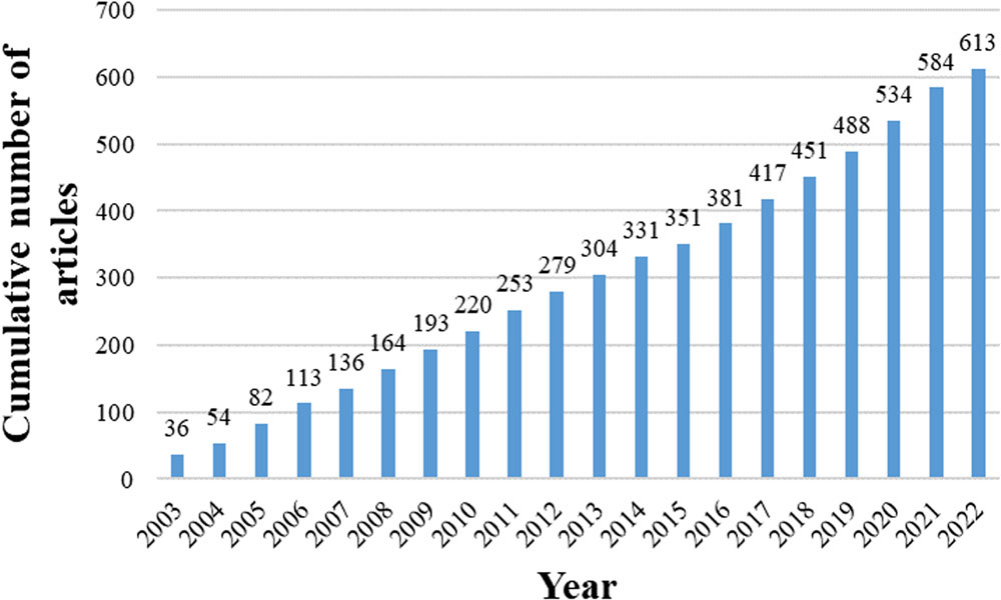
Global number of annual publications related to hydrocephalus research from 2003 to 2022. [Color figure can be viewed at wileyonlinelibrary.com]

A total of 613 records from the WoSCC database between 2003 and 2022 were closely related to fetal hydrocephalus. After using CiteSpace to remove duplication, no duplicate literature was found, and 613 articles were finally included. We assessed annual publication and trends over the years (Figure 2). It showed that the number of articles related to fetal hydrocephalus between 2005 and 2014 is stabilized at around 28, and the number of papers published in 2018–2021 gradually increased, reaching a maximum of 50 in 2021.

### 3.2 National contribution

**Figure 3:**
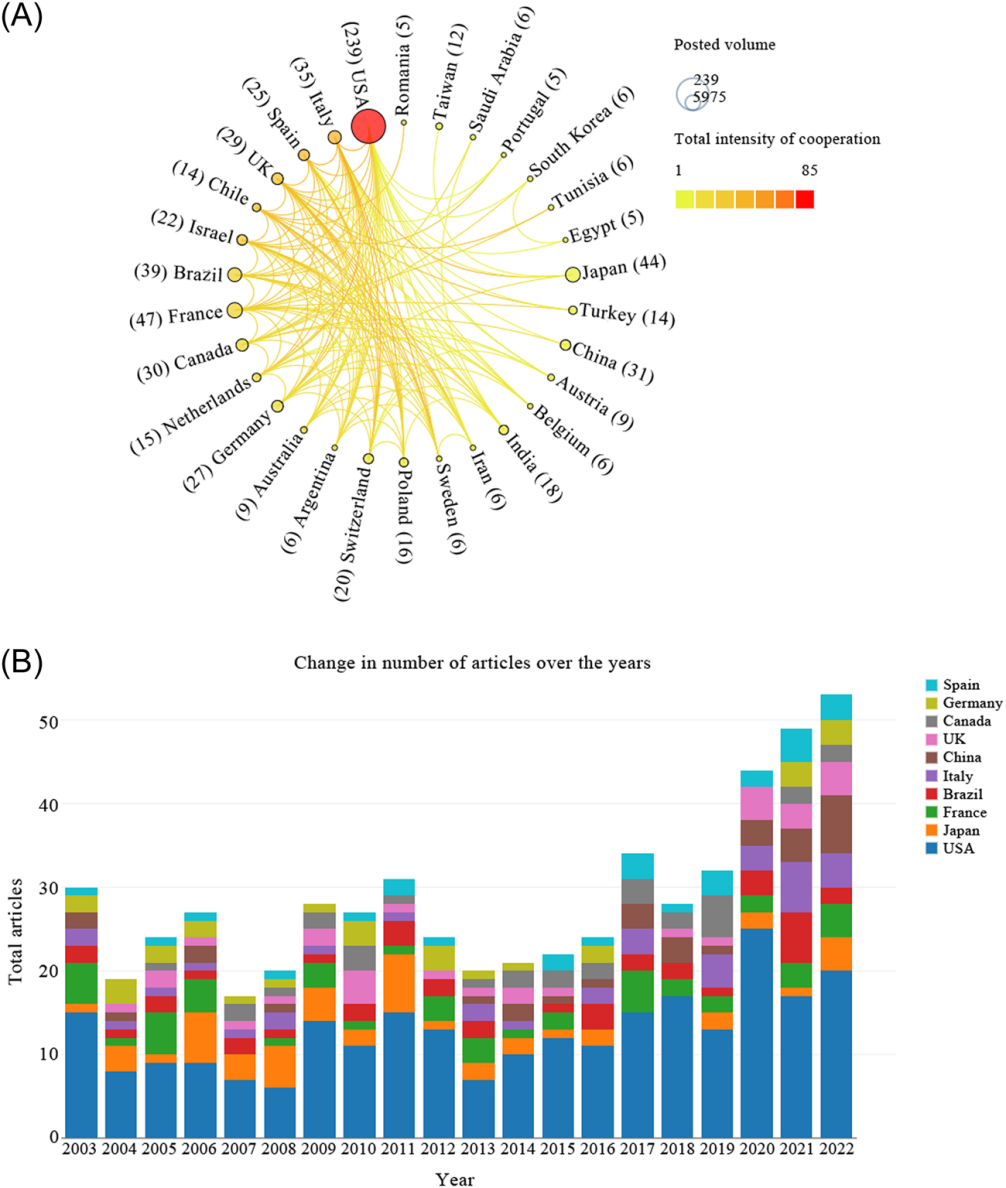
Country contributions on fetal hydrocephalus. (A) Network of country distribution based on VOSviewer articles. The size of the node indicates the influence of the country, and the thickness of the connecting line between the two nodes indicates the intensity of collaboration. (B) Annual publications from top 10 countries. [Color figure can be viewed at wileyonlinelibrary.com]

A total of 66 countries published studies related to fetal hydrocephalus. Among these countries, 30 countries were closely related to each other. Visual analysis showed that the United States has always been the center of fetal hydrocephalus research in the world with a total of 239 published papers, far exceeding other countries. France and Japan, with a total of 47 and 44 papers respectively, are also among the top countries in the world. However, published papers from other countries on hydrocephalus fetal hydrocephalus still need to be improved. The publication networks between different countries reveal the collaborations between different countries. Notably, researchers from the United States collaborate extensively with their counterparts from other nations, exhibiting the strongest intensity of cooperation. It showed that countries closely related to United States included Japan, France, and Brazil, which also have significant influence in this field, second only to the United States (Figure 3A).

Using Bibliometric Online Analysis Platform, we analyzed the number of published papers between countries over years. From 2003 to 2022, the United States consistently led other countries in the number of articles published in the study on hydrocephalus. France is also ahead of most countries in the study of fetal hydrocephalus, and its contribution in 2003, 2005, 2013, and 2017 is second only to that of the United States. Italy and Spain have also made great contributions to the study of fetal hydrocephalus. China also ranks in the top 10 in the number of published articles, and this number has gradually increased in recent years, ranking second only to the United States in 2022. In addition to China, countries in East Asia, such as Japan, have gradually increased the number of articles published on the study of fetal hydrocephalus, and even surpassed France to rank second in the world in 2006 and 2011. (Figure 3B).

### 3.3 Journal analysis

**Figure 4:**
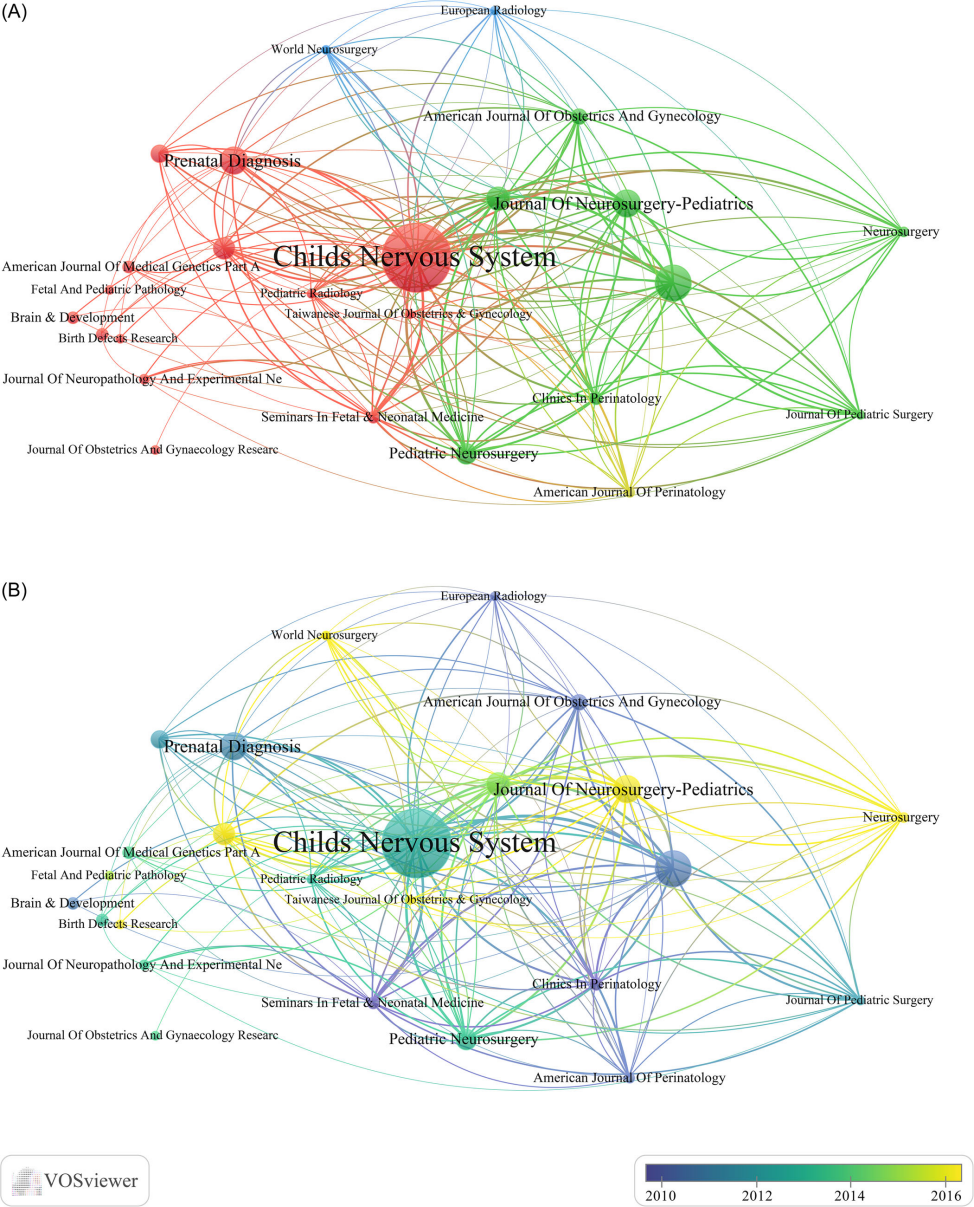
Journal contributions on fetal hydrocephalus. (A) Networks of journal distribution based on VOSviewer. The four colors represent different fields of fetal hydrocephalus research. (B) Journal contributions related to hydrocephalus published from 2003 to 2022 over years. The size of the node indicates the influence of the journal, and the thickness of the connecting line between the two nodes indicates the closeness of the relationship. The color of the line indicates journal contributions over years, and yellow indicates the latest contributions. [Color figure can be viewed at wileyonlinelibrary.com]

Researchers used the VOSviewer to analyze the influence of journals. The 38 journals that published at least four papers were included in the network diagram and divided into four clusters. As represented in the co‐occurrence network (Figure 4A), the identified references were divided into four clusters. The red cluster was the largest, which was for diagnostics journals. The second is green cluster, which is a journal of pediatrics; Finally, the blue cluster is for surgical journals and the yellow cluster is for obstetrics and gynecology journals. *Childs Nervous System* is the most influential journal. *Childs Nervous System* and *Journal Of Neurosurgery‐Pediatrics*, *World Neurosurgery*, *American Journal Of Perinatology*, are the most important journals in these clusters, respectively (Figure 4A). According to the publication volume of different journal on fetal hydrocephalus in different years, it showed that *Childs Nervous System* and *Prenatal Diagnosis* published the most of articles about fetal hydrocephalus before 2014, making them the most influential journal in related field. Recently, journals such as *World Neurosurgery*, *Journal of Neurosurgery‐Pediatrics* and *Neurosurgery* emerged as vital journals for increased publication since 2014. Around 2012, *Childs Nervous System* published a large number of all studies on fetal hydrocephalus, which was significantly higher than that of other journals. This trend continued, and by 2022, the contribution of Childs Nervous System on fetal hydrocephalus remained far greater than that of any other journal (Figure 4B).

### 3.4 Author contribution

**Figure 5:**
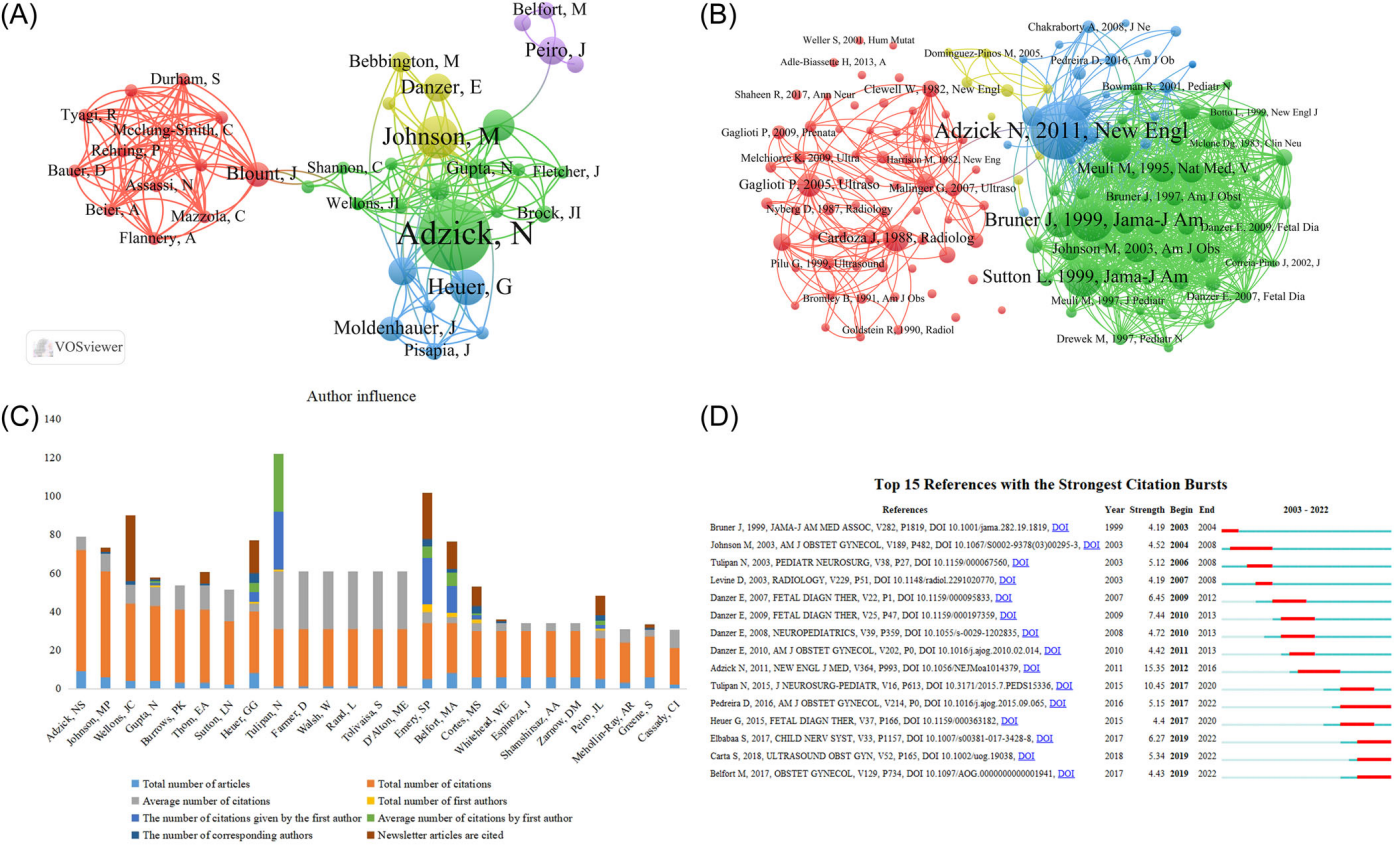
Authors' contribution map. (A) Clustering analysis of co‐citation network titles based on citation space. (B) Network diagram of publications cited. (C) Total number of articles, total number of citations, average number of citations, total number of one article, the number of citations of one article, the average number of citations of one article, the number of correspondent authors and the number of citations of correspondent articles in the top 25 authors' influence ranking from 2003 to 2022. (D) The first 15 most frequently cited references based on citation space. [Color figure can be viewed at wileyonlinelibrary.com]

Article publication and citation are important indicators of author influence. We used VOSviwer and CiteSpace to analyze author contributions. A total of 3122 authors were involved in the fetal hydrocephalus study, among which 124 authors published at least three articles, who were subsequently included in the authors' network diagram and divided into five groups, with Peiro J., Adzick N. S., Johnson M. P., Heuer G., and Blount J. at the center of these groups respectively. Adzick N. S. is the most effective and influential author in the field of fetal hydrocephalus research with the most published articles and the most cited articles (Figure 5A, Table 1), among which “Adzick et al.^15^” was the most cited reference (Figure 5B, Table 2). Most of the active collaboration in fetal hydrocephalus research is conducted among the same group of authors, such as Heuer G. and Moldenhauer J., Blount J. and Mazzola C. A., and Belfort M. and Peiro J. (Figure 5A). Overall, Adzick, N. S. had the largest total article count and total citation counts, followed by Johnson M. P., while for mean citation counts, Tulipan N., Farmer D., Walsh W., Rand L., Tolivaisa S., D'Alton M. E. far exceeded other authors, reached 30 times, a citation times (Figure 5C, Table 1). We analyzed the references using the burst detection feature in CiteSpace. The first 15 most cited references are shown in Figure 5D and Table 2. It is worth noting that the reference “Adzick et al.^15^” was cited the most, with a citation strength of 15.35 and cited 105 times (Figure 5D, Table 2).

**Table 1:** Hydrocephalus author influence.

Author	Total number of citations	Total number of articles	Average number of citations	Total number of first authors	The number of citations given by the first author	Average number of citations by first author	The number of corresponding authors	Newsletter articles are cited
Adzick, NS	63	9	7	0	0	0	0	0
Johnson, MP	55	6	9.17	0	0	0	1	2
Wellons, JC	40	4	10	0	0	0	2	34
Gupta, N	39	4	9.75	1	1	1	1	1
Burrows, PK	38	3	12.67	0	0	0	0	0
Thom, EA	38	3	12.67	0	0	0	1	6
Sutton, LN	33	2	16.5	0	0	0	0	0
Heuer, GG	32	8	4	1	5	5	5	17
Tulipan, N	30	1	30	1	30	30	0	0
Farmer, D	30	1	30	0	0	0	0	0
Walsh, W	30	1	30	0	0	0	0	0
Rand, L	30	1	30	0	0	0	0	0
Tolivaisa, S	30	1	30	0	0	0	0	0
D'Alton, ME	30	1	30	0	0	0	0	0
Emery, SP	29	5	5.8	4	24	6	4	24
Belfort, MA	26	8	3.25	2	14	7	2	14
Cortes, MS	24	6	4	2	2	1	4	10
Whitehead, WE	24	6	4	0	0	0	1	1
Espinoza, J	24	6	4	0	0	0	0	0
Shamshirsaz, AA	24	6	4	0	0	0	0	0
Zarnow, DM	24	6	4	0	0	0	0	0
Peiro, JL	21	5	4.2	1	2	2	3	10
Mehollin‐Ray, AR	21	3	7	0	0	0	0	0
Greene, S	21	6	3.5	0	0	0	1	2
Cassady, CI	19	2	9.5	0	0	0	0	0

**Table 2:** Top 15 most cited references in the field of hydrocephalus.

Cited references	Citations	Total link strength
Adzick et al. (2011)	102	1005
Bruner et al. (1999)	76	1036
Sutton (1999)	66	910
Tulipan (2003)	50	665
Rintoul (2002)	48	711
Meuli (1995)	44	677
Cardoza (1988)	41	345
Mclone (1989)	41	413
Heffez (1990)	40	661
Johnson (2003)	40	617
Adzick (1998)	39	665
Gaglioti (2005)	36	291
Paek (2000)	34	569
Meuli (1995)	31	520
Tulipan (2015)	31	290

### 3.5 Key words

**Figure 6:**
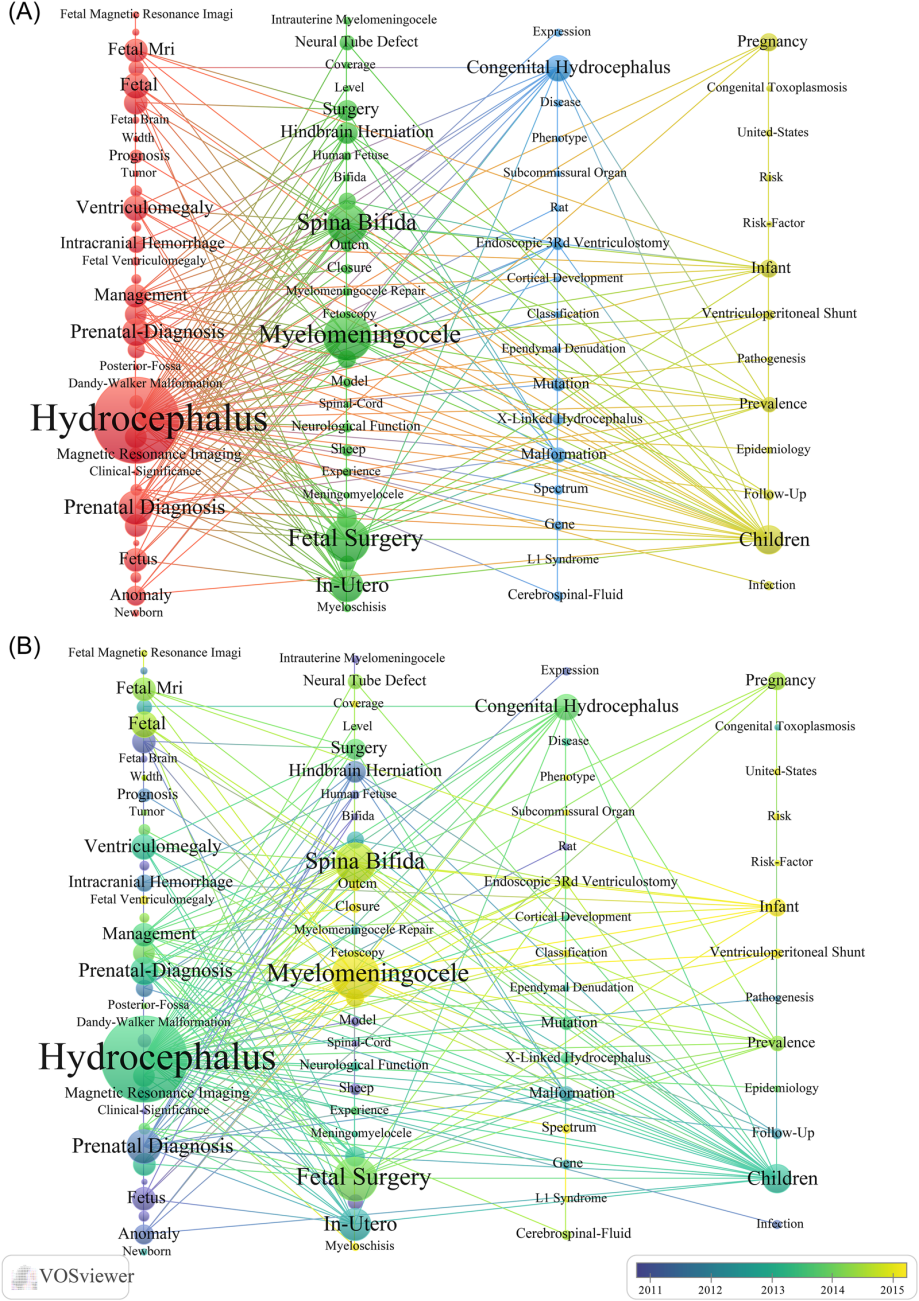
Keywords mapping in hydrocephalus research. (A) Keyword clustering analysis based on VOSviewer. (B) Chronological order of keyword trends based on VOSviewer. [Color figure can be viewed at wileyonlinelibrary.com]

To track the development trends and hot spots in the research field of fetal hydrocephalus, we performed keyword co‐occurrence analysis using VOSviewer. As represented in the co‐occurrence network, the identified keywords can be divided into four clusters, and the keywords clustered in the red area include the main terms related to hydrocephalus and prenatal diagnosis, with the following keywords appearing frequently: “Hydrocephalus,” “Pre‐Natal Diagnosis,” “Fetal,” “Ventriculomegaly,” and “Ultrasound.” The keywords aggregated in the green area mainly describe Myelomeningoele, and the frequently‐occurring keyword is “Surgery,” “Myelomeningoele,” “Spina Bifida,” “Fetal Surgery” and “In‐Utero.” The key words clustered in the blue area were mainly “Congenital Hydrocephalus.” “Endoscopic 3rd ventriculostomy.” The yellow region cluster is mainly a cluster about the epidemiology of the disease (Figure 6).

We also performed a visual analysis of the fetal hydrocephalus study‐related keywords over years using VOSviewer, as shown in Figure 6 where each node represented a keyword, and the color indicated the year that the keyword mostly appeared in the analyzed data set. Most of the keywords in the fetal hydrocephalus study were appeared in 2013 was the most, and the number of new keywords that appeared gradually decreased with the increase of the year. In conclusion, at the beginning of the study, the key words were mainly related to child and prenatal diagnosis. In recent years, there have been studies related to meningocele and fetal surgery.

## 4 DISCUSSION

Bibliometric analysis is an excellent method that provides experienced researchers with systematic and visual knowledge structures and helps new scientific researchers to get the general trends in their field of study.^16^ Herein, we perform the bibliometric knowledge analysis on the field of fetal hydrocephalus during the last two decades. Bibliometrics is now widely used in various fields of global research to help researchers gain an intuitive and systematic understanding of a field and identify important scientific achievements and future research hot spots. In this study, the published journals, publication date, institution, country, author, and keywords of hydrocephalus were analyzed using bibliometric and visual methods, to reveal the current status of hydrocephalus research in the world. We found that the United States was identified as the most productive country. *Children nervous system* has become the most frequently cited periodical, and Adzick N. S. is recognized as the most productive author. We have also identified various research hotspots in the past so far, mainly focusing on fetal surgery, spina Bifida and in‐utero. It is expected that the follow‐up research will focus on the effects of myelomeningole on fetal hydrocephalus.

### 4.1 General trends

The change in annual output is generally considered to be an important indicator of the development process of a research field.^17^, ^18^ According to data obtained from the WoSCC database, a total of 613 articles have been published over the past 20 years. In the past studies, the number of publications on fetal hydrocephalus was gradually increased, indicating that this field was the current research focus. The research on fetal hydrocephalus has developed rapidly since 2020. The small number of publications in 2022 may be due to the timeliness of this study. Overall, we anticipate that the growth trend in fetal hydrocephalus publications may continue in the coming years. The United States is the leader in fetal hydrocephalus research, with France second, both in number of publications and total citation. The United States produced almost half of all publications between 2003 and 2022, and publications from other countries have grown over the years. In addition to the United States and France, we found that Japan is also very productive in this area, ranking second in global publications in 2006 and 2011. We observed some positive cooperation between different countries/regions, most of which are concentrated in North American and European countries, such as United States–Brazil, United States–Canada, and United States–Chile, with United States as the main cooperation center. It is worth noting that since 2019, China's research publications on fetal hydrocephalus have been increasing, and they have reached the second place in the world by 2022. We observed that China has also become a potential large country for fetal hydrocephalus research, which means that research related to fetal hydrocephalus has attracted worldwide interest.

### 4.2 Contribution of journals

Based on journal analysis, as of December 31, 2022, *Childs Nervous System*, *Journal Of Neurosurgery‐Pediatrics*, and *American Journal Of Perinatology* had published the most fetal hydrocephalus‐related manuscripts. Other influential medical journals such as *obstetrics and gynecology* have also published papers related to fetal hydrocephalus, which means that fetal hydrocephalus has attracted extensive attention from researchers in various countries around the world. Publishing excellent papers can improve the academic quality and influence of these journals.

### 4.3 Author contribution

Evaluating the contributions of influential authors in the field of science can help researchers better understand this field and open up new directions.^19^ Among the 3122 authors who have contributed to the study of fetal hydrocephalus, Adzick N. S. is the most contributing author in this field. He has published nine manuscripts in total, and the number of citations of the articles also ranks first with 63 times. Johnson M. P. has published six manuscripts related to fetal hydrocephalus, and the number of citations is second only to Adzick N. S. It is gratifying that some Asian authors have also actively participated in the cooperative cluster of fetal hydrocephalus research.

### 4.4 Research hotspots of hydrocephalus

As for the related key words involving hydrocephalus, the most common keywords are “Prenatal Diagnosis,” “Myelomeningocele,” “Hydrocephalus,” “Neural Tube Defect,” and so forth.

Prenatal diagnostic studies mainly focus on fetal magnetic resonance imaging (MRI) and ultrasound studies. Diagnosis in utero by ultrasound gives early opportunity for treatment of fetal hydrocephalus.^20^ Fetal hydrocephalus is usually studied after 17 weeks of gestation,^21^ and the results of prenatal examinations can predict morbidity and mortality with relative accuracy.^22^ Given that a large number of terminations were performed at the time of prenatal diagnosis, it is likely that the incidence of fetal hydrocephalus was indeed higher than these rates.^23^, ^24^, ^25^ For the prenatal period, ventricle width <10 mm is considered normal, while ventricular enlargement was classified as mild to moderate at 10–15 mm and as severe when measurements exceeded 15 mm.^26^ Distinguishing between fetal ventricular enlargement and hydrocephalus is complex but crucial. Ventricular hypertrophy may also be the result of fetal brain atrophy or hypoplasia and malformations associated with agenesis of the corpus callosum. In hydrocephalus, ventricular enlargement is usually accompanied by hypertension, and the subarachnoid space is usually reduced. In contrast, the choroid plexus and subarachnoid space are preserved during ventricular enlargement. In addition, there is evidence that diseases diagnosed prenatally are more severe than those diagnosed after birth, especially severe ventricular enlargement.^21^ So, it can be concluded that the earlier the pathological condition appears, the more severe it is.

Green cluster is a clinical treatment study, from a neurosurgical point of view. Myelomeningocele is the most significant complication associated with hydrocephalus, necessitating shunt therapy in 75%–80% of affected patients. However, recent advances, such as intrauterine myelomeningocele closure and endoscopic third ventriculostomy combined with choroid plexus cauterization (ETV‐CPC), are increasingly reducing the need for shunt therapy. The presentation of hydrocephalus varies from patient to patient and throughout the life span.^27^ From a neurosurgical point of view, the treatment of hydrocephalus is “base zero” for the treatment of spina bifida.^28^, ^29^ Subtle declines in ventricular function or inadequate drainage/decompression can stress an already compromised nervous system and promote the amplification of downstream problems.^27^ The clinical treatment of fetal hydrocephalus mainly relies on surgery. ETV‐CPC are low‐cost, safe, and promising interventions for the treatment of spina bifida related hydrocephalus (SBHCP).^30^

Green clusters and blue clusters are clinical treatment related class studies, and yellow clusters are prognostic class studies, which are relatively rare. In this review, they are discussed together. Well‐known genetic disorders and cytogenetic abnormalities are associated with congenital hydrocephalus. The most common cytogenetic abnormalities in symptomatic hydrocephalus include (Mosaic) trisomies 9, 9p, 13, and 18 and (Mosaic) triploidy,^31^ and the most related genetic disorder is X‐linked hydrocephalus, as indicated by the keywords in the blue cluster. Today, congenital hydrocephalus is routinely and successfully treated after birth with a shunt or endoscopic third ventriculostomy (ETV). However, in the recent past, hydrocephalus was associated with dismal outcomes and high mortality.^32^ Given that postpartum treatment may be too late,^15^ efforts have been made to provide some effective prenatal fetal treatments to prevent or halt the progression of hydrocephalus and its secondary neurological deterioration before birth. This led to the development of intrauterine fetal surgery. As early as the early 1980s, prenatal shunt procedures such as serial cephalocentesis and Ventriculo‐amniotic shunt were tried to contain fetal ventricular enlargement.^33^ However, subsequent studies have shown that the prognosis of this approach is unsatisfactory.^34^, ^35^ These early intrauterine treatment attempts are labeled and stigmatized for poor clinical outcomes and high mortality rates, worse than in patients undergoing shunt surgery in the postpartum period. In 1986 Michejda et al.^36^ commented that the methods used for hydrocephalus shunting at that time were inadequate, suggesting that the open technique via hysterotomy may be a more effective method of inserting an appropriate ventriculo‐amniotic shunt (VAS) and may become the preferred treatment for carefully selected progressive fetal hydrocephalus. A subsequent series of clinical retrospective studies also confirmed the relative reliability of this method.^37^ ETV is one of the treatments for fetal. At present, there is a study to explore the complications and mortality of ETV in the treatment of fetal hydrocephalus. The research subjects were 40 infants with fetal hydrocephalus. The results showed that 19 infants needed a second CSF shunt 6 months after operation. The failure rate of infants less than 3 months is obviously higher, and all infants eventually underwent ventriculoperitoneal shunt. The incidence of ETV site uplift, one of the most common postoperative complications, was 20%. Additionally, 15% of patients presented clinical signs of meningitis after ETV, 10% experienced CSF leakage at the ETV site, 7.5% developed subdural effusion, and 17.5% of the patients died on average 22 days postoperation. The incidence of ETV site uplift (one of the most common complications after operation) is 20%. Additionally, 15% of patients presented clinical signs of meningitis after ETV, 10% experienced CSF leakage at the ETV site, 7.5% developed subdural effusion, and 17.5% of the patients died on average 22 days postoperation.^38^ Of course, this also promoted the development of new technology. Using a recently developed novel sheep model of fetal obstructive hydrocephalus,^39^ in which a bio‐glue is injected into the fetal cisterna, it demonstrated that endoscopic navigation in the fetal ventricle is possible. This implies that more animal studies are needed to lay the foundation for successful clinical application of the new technique.

Standard treatment is currently provided postpartum through ventriculoperitoneal shunt and ETV, among others. These postpartum treatments can only partially restore the nerve injury and it is mainly alleviated by reducing intracranial pressure.^40^, ^41^ The best treatment of infant hydrocephalus has not been clearly defined. Recently, advances have been made in the fields of neuroendoscopy and shunt hardware, but the treatment of infant hydrocephalus remains one of the most difficult challenges faced by neurosurgeons.^42^, ^43^, ^44^, ^45^, ^46^, ^47^, ^48^, ^49^ New medical techniques can change the way fetal hydrocephalus is treated and its consequences, as a complement to cerebrospinal fluid shunts for shunting procedures. Cell transplantation therapies for brain diseases have been the subject of numerous publications. A number of recent studies have begun to lay the foundation for cellular therapy for fetal onset fetal hydrocephalus, underscoring potential cells for brain transplantation including pluripotent neural stem cells, mesenchymal stem cells, genetically engineer stem cells, choroid plexus cells, and subcellular organ cells.

In general, fetal hydrocephalus needs to be detected and treated in time, otherwise it will cause irreversible damage. Recent improvements in imaging, patient selection, and fetal surgical techniques in the intrauterine management of the fetus suggest that this topic warrants further evaluation. The emerging medical technology may be a good way to treat hydrocephalus, indicating that the clinical treatment of hydrocephalus needs a lot of basic research to improve the prognosis of these fetal and neonatal patients.

In this study, we extracted the relevant publications on fetal hydrocephalus from the WoSCC database and fully analyzed the current hot trends, but there are still some limitations. For example, we only analyze English‐language publications, resulting in documents of non‐English quality that may be ignored. Follow‐up cooperation with researchers from other countries should therefore be initiated to obtain more in‐depth and comprehensive analysis results.

## 5 CONCLUSION

In summary, this study comprehensively summarized and analyzed the global research trends of fetal hydrocephalus research. In recent years, the number of high‐quality publications related to this field has increased significantly, and fetal hydrocephalus has a good development prospect in the diagnosis and treatment of fetal period.

## AUTHOR CONTRIBUTIONS

Qian Li carried out conceptual design, data collection and data analysis. Zheyu Song, Chenyang Zhai, and Wenxue Zhao were responsible for data collection, compilation and data analysis. Sajid Hussain wrote the manuscript. Shunwu Xiao carried out conceptual design and administrative support, manuscript writing and final approval of the manuscript. All authors have read and approved the final content of this manuscript.

## CONFLICT OF INTEREST STATEMENT

The authors declare no conflict of interest.

## ETHICS STATEMENT

Not applicable.

## Data Availability

The data sets for this study are available from the corresponding author upon reasonable request.
